# Potential Associations Between CT-Derived Muscle Indices and Clinical Outcomes in Acute Pancreatitis

**DOI:** 10.3390/medicina62010054

**Published:** 2025-12-27

**Authors:** Selma Özlem Çelikdelen, Zeynep Keskin, Tevhide Şahin, Korhan Kollu, Muhammet Cemal Kizilarslanoglu

**Affiliations:** 1 Department of Internal Medicine, Konya City Hospital, University of Health Sciences Türkiye, 42020 Konya, Türkiye; 2 Department of Radiology, Konya City Hospital, University of Health Sciences Türkiye, 42020 Konya, Türkiye; zkeskin@ymail.com; 3 Division of Gastroenterology, Department of Internal Medicine, Konya City Hospital, University of Health Sciences Türkiye, 42020 Konya, Türkiye; sahintevhide@gmail.com; 4 Division of Intensive Care Medicine, Department of Internal Medicine, Konya City Hospital, University of Health Sciences Türkiye, 42020 Konya, Türkiye; korhankollu@gmail.com; 5 Division of Geriatrics, Department of Internal Medicine, Konya City Hospital, University of Health Sciences Türkiye, 42020 Konya, Türkiye; drcemalk@yahoo.com.tr

**Keywords:** acute pancreatitis, psoas muscle index, paravertebral muscle index, sarcopenia

## Abstract

*Background and Objectives:* Acute pancreatitis (AP) is one of the most common gastrointestinal emergencies worldwide. Early identification of high-risk patients is essential to improve outcomes. Computed tomography (CT)-derived muscle mass indices, such as the psoas muscle index (PMI) and paravertebral muscle index (PvMI), have recently emerged as potential prognostic markers reflecting both nutritional and inflammatory status. This study aimed to investigate the relationship between CT-derived PMI and PvMI with disease severity, complications, and intensive care unit (ICU) requirement in patients with acute pancreatitis, and to evaluate their prognostic value across age- and sex-specific subgroups. *Materials and Methods:* This retrospective study included 179 patients hospitalized with AP between January 2023 and February 2025. The psoas muscle area (PMA) and paravertebral muscle area (PvMA) were measured at the L3 vertebral level on CT scans and normalized to height squared to calculate the PMI and PvMI levels. Additionally, patients were classified as having low or normal PMA and PvMA levels based on cutoff values from the existing literature. Clinical, biochemical, and outcome data—including disease severity, complications, and ICU requirement—were analyzed. Subgroup analyses were performed by sex and age (≥65 years). Logistic regression and ROC analyses were used to identify independent predictors and optimal cutoff values. *Results*: Overall, complications developed in 39.7% of patients, and ICU admission was required in 11.2%. The PMI levels were significantly correlated with albumin, hemoglobin, and inflammatory marker levels. In women, the PMI was independently associated with complicated AP (adjusted OR = 0.655, *p* = 0.018). In patients ≥65 years, the PvMI level was independently associated with ICU requirement (adjusted OR = 0.780, *p* = 0.047). The ROC analysis identified PMI ≤ 4.04 cm^2^/m^2^ as the optimal cutoff for predicting complicated AP (AUC = 0.641, *p* = 0.049), and PvMI ≤ 18.88 cm^2^/m^2^ for predicting ICU need (AUC = 0.684, *p* = 0.020), with moderate discrimination. *Conclusions*: CT-derived muscle indices might be associated with disease severity and adverse outcomes in AP, particularly among older (≥65 years) and female patients. PMI and PvMI may serve as practical prognostic markers to identify high-risk patients early, enabling timely nutritional and supportive interventions. Validation in larger prospective cohorts is warranted.

## 1. Introduction

Acute pancreatitis is one of the most common causes of gastrointestinal emergency admissions worldwide, with an incidence ranging from 13 to 45 cases per 100,000 population [1]. While the majority of cases follow a mild and self-limiting course, approximately 20–30% progress to moderate or severe disease, which is associated with local or systemic complications, intensive care requirement, and increased mortality. In severe cases of AP, mortality rates can reach 15–30% [2]. Therefore, predicting disease severity early and identifying high-risk patients are critical for planning treatment strategies and improving prognosis. For this purpose, several prognostic scoring systems have been developed. Methods such as the Ranson criteria, APACHE II, and BISAP are widely used to assess disease severity [3,4]. However, most of these scoring systems require repeated evaluation of clinical and laboratory parameters in the following days, may be time-consuming in practice, and offer limited predictive value in the early phase of the disease. Therefore, there remains a need for easily accessible, practical, and early-applicable additional prognostic markers.

In recent years, CT-based muscle mass measurements have gained importance as prognostic markers across various disease groups. Quantitative assessment of muscle mass using radiological methods can provide additional predictive information on the clinical course by reflecting both nutritional status and the inflammatory response. Sarcopenia is a syndrome characterized by loss of muscle mass and/or muscle function due to age or disease. It interacts bidirectionally with systemic inflammation and is associated with a poor prognosis [5]. In the assessment of sarcopenia, CT measurement of the psoas muscle area at the L3 vertebral level is one of the most commonly used and reliable methods [6]. Previous studies have shown that sarcopenia in patients with chronic pancreatitis is associated with increased hospitalization rates, poorer surgical outcomes, reduced survival, and adverse prognosis [7,8]. Similarly, in patients with AP, possible associations between muscle mass parameters and disease course have also been examined. However, the available evidence on this subject remains limited and heterogeneous, although interest in this field has grown considerably in recent years [9]. Given these limitations, further research is warranted to clarify the prognostic significance of muscle indices in AP. In this context, the present study aimed to evaluate the relationship between CT-derived psoas and paravertebral muscle indices and disease severity, development of complications, and the need for intensive care in patients with acute pancreatitis. In addition, by performing subgroup analyses according to age and sex, we sought to provide a more detailed understanding of the association between muscle mass and clinical outcomes.

## 2. Materials and Methods

### 2.1. Study Design

This retrospective study reviewed the electronic medical records of patients hospitalized with a diagnosis of AP in the Departments of Internal Medicine and Gastroenterology at Konya City Hospital between January 2023 and February 2025. During this period, a total of 313 patient files were screened.

### 2.2. Pancreatitis Classification and Patient Selection

The diagnosis of acute pancreatitis was established according to the 2012 Revised Atlanta Classification. Accordingly, the diagnosis was confirmed by the presence of at least two of the following criteria: typical abdominal pain, serum amylase or lipase levels ≥ 3 times the upper limit of normal, and imaging findings consistent with acute pancreatitis. Disease severity was categorized according to the same classification as mild, moderately severe, and severe acute pancreatitis. Mild AP was defined by the absence of organ failure and complications, whereas moderately severe AP was characterized by transient organ failure (<48 h) and/or local or systemic complications. Severe AP was defined as persistent organ failure (≥48 h). According to the Revised Atlanta Classification, local complications include acute peripancreatic fluid collection, acute necrotic collection, pancreatic pseudocyst, and walled-off necrosis. In contrast, systemic complications are defined as organ dysfunction or exacerbation of pre-existing comorbidities [10]. The presence of any of these complications was described as complicated pancreatitis. In addition, patients were classified according to whether they required intensive care during follow-up.

**Figure 1:**
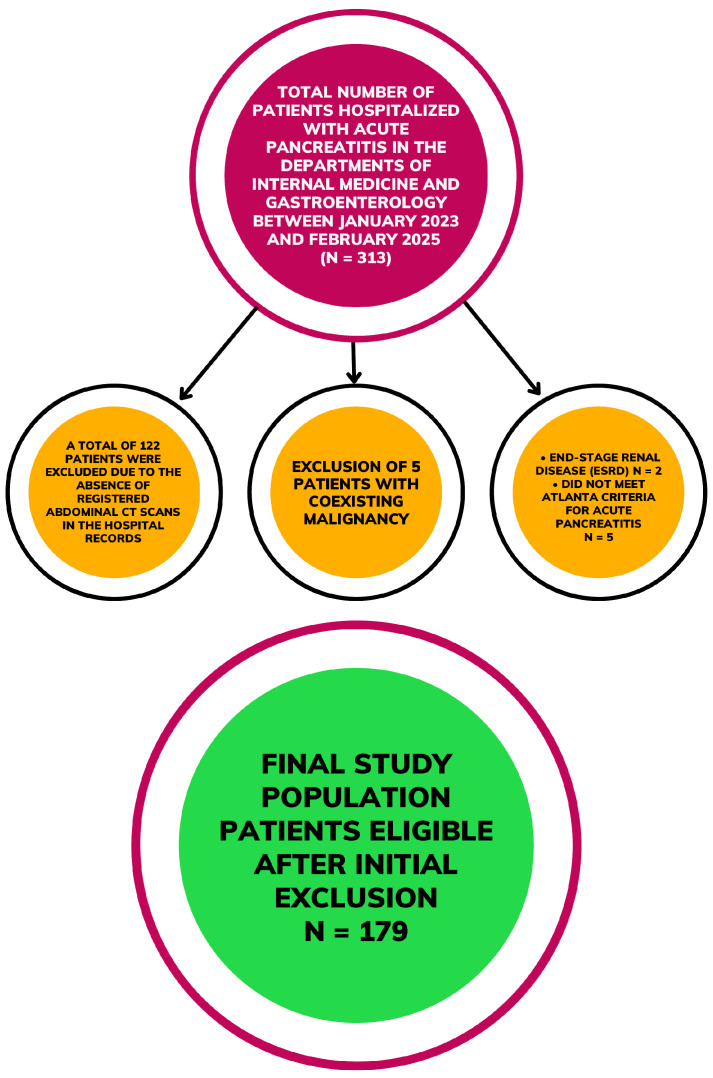
Flowchart of patient selection and study population.

During the study period, 313 patients with AP were screened. Of these, 122 had no available CT imaging records in the hospital system, five did not meet the diagnostic criteria of the Revised Atlanta Classification, 5 had coexisting malignancy, and 2 had end-stage renal disease. After applying these exclusion criteria, 179 patients were included in the final analysis (Figure 1).

### 2.3. Patient’s Characteristics, Laboratory Measurements, and CT Imaging

Data on patients’ age, sex, height, weight, history of acute pancreatitis, pre-existing comorbidities, etiology and severity of AP, length of hospital stay, need for intensive care, and complications were retrieved from medical records. Laboratory findings at admission included complete blood count parameters and biochemical tests (alanine aminotransferase [ALT], aspartate aminotransferase [AST], alkaline phosphatase [ALP], γ-glutamyltransferase [GGT], bilirubin levels, lipid profiles, and serum electrolytes). In addition, procalcitonin and C-reactive protein (CRP) levels measured within 48–72 h after admission were recorded. Abdominal CT scans obtained during hospitalization for suspected AP were evaluated by a radiologist specialized in abdominal imaging who was blinded to all clinical, laboratory, and outcome data. At the L3 vertebral level, the right and left psoas and paravertebral muscle areas were manually delineated on axial slices. These areas were summed to calculate the total muscle cross-sectional area, which was then normalized by height squared to derive the PMI and PvMI [6,11]. Also, PMA and PvMA levels were dichotomized (low or normal) according to cutoffs reported in the literature (low PMA was defined as 11.50 cm^2^ for men and 8.22 cm^2^ for women; low PvMA was defined as 52.9 cm^2^ for men and 33.2 cm^2^ for women) [12,13].

Patients’ Chronic disease status was evaluated using the Charlson Comorbidity Index (CCI), a validated scoring system that quantifies the burden of comorbidities based on predefined chronic conditions. The CCI was calculated according to the updated methodology by Quan et al. [14], and the list of comorbidities and scoring details is provided in Supplementary Table S1.

The collected data were then analyzed with respect to the severity, complications, and intensive care unit (ICU) requirements of acute pancreatitis.

### 2.4. Statistical Analyses

In this study, statistical analyses were performed using the Statistical Package for the Social Sciences (SPSS) software, version 26.0 (IBM Corp., Armonk, NY, USA). First, categorical and numerical variables were examined in detail. Categorical variables were expressed as numbers and percentages. In contrast, numerical variables were presented according to their distribution characteristics: those with normal distributions were reported as mean ± standard deviation, and those without normal distributions were reported as median (minimum–maximum). Comparisons between groups were performed using the Chi-square or Fisher’s Exact test, Student’s *t*-test, and the Mann–Whitney *U* test. To examine the correlations among numerical variables, the Spearman test (ρ coefficient) was applied. The associations between complicated pancreatitis and ICU requirement, and PMI and PvMI values, were analyzed using univariate and multivariate logistic regression models. Similarly, optimal cutoff values for PMI and PvMI to distinguish complicated pancreatitis and the need for intensive care were determined using receiver operating characteristic (ROC) analyses. A *p*-value of <0.05 was considered statistically significant.

## 3. Results

### 3.1. General Characteristics and Clinical Features

The median age of the 179 patients in the study was 58 years, and 53.1% were female. A total of 35.8% of the patients were aged 65 years or older (n = 64). In the overall patient group, the incidence of complications was 39.7% (n = 71), and the frequency of ICU requirement was 11.2% (n = 20). The in-hospital mortality rate was 1.7% (n = 3) in the overall cohort, whereas it was 4.2% among patients with complicated disease and 15% among those requiring ICU care (*p* < 0.001). The mean age was significantly higher among patients with complicated pancreatitis and those requiring ICU care (63 and 68 years, respectively; *p* = 0.05). The mean hospital stay was significantly longer in patients with complicated pancreatitis than in those without complications (mean: 10 days, *p* < 0.05). Mean CRP levels were significantly higher in patients with complicated pancreatitis and those requiring ICU care (23.42 mg/L and 47.19 mg/L, respectively; *p* < 0.005), as were mean procalcitonin levels (0.38 µg/L and 3 µg/L, respectively; *p* < 0.001). Mean albumin and calcium levels were significantly lower in patients requiring ICU care (37.5 g/L and 8.25 mg/dL, respectively; *p* < 0.05). In the overall cohort, the CCI score was significantly higher in patients requiring ICU care (mean score: 1 [0–4]; *p* < 0.05). No significant differences in PMI or PvMI values were observed between patients with and without complicated pancreatitis or ICU requirement (all comparisons, *p* > 0.05). When patients were categorized into low PMA and normal PvMA groups, no significant differences were observed in the rates of complications or ICU requirement in the overall cohort, as well as in sex-specific subgroups and the older (≥65 years) population (all *p* > 0.05). The general characteristics of the patients and their comparisons according to the presence of complicated pancreatitis and ICU requirement are presented in Table 1.

### 3.2. Correlation Analyses Between Muscle Indices and Clinical–Biochemical Parameters

In correlation analyses performed for the entire patient group, a weak but statistically significant positive correlation was observed between PMI and albumin levels, and a weak negative correlation between PMI and CRP levels. PvMI showed weak, statistically significant negative correlations with WBC, hemoglobin, and creatinine levels (Table 2).

### 3.3. Correlation Analyses Stratified by Sex

In female patients, PMI showed statistically significant negative correlations with WBC (moderate), neutrophil count, and triglyceride levels, and a positive correlation with albumin levels. PvMI was negatively correlated with ALT, GGT, and creatinine levels. In male patients, PMI was positively correlated with amylase, lipase, and glucose levels, and negatively correlated with CRP. In addition, PvMI showed positive correlations with amylase, lipase, and ALP levels (all *p* < 0.05). Overall, most of these correlations were of weak magnitude (Table 2).

### 3.4. Correlation Analyses in Patients Aged 65 Years and Older

In patients aged 65 years and older, PvMI showed negative correlations with creatinine (moderate) and ALT (weak). In the subgroup of older female patients, additional negative correlations were observed between PvMI and GGT, AST, and ALT (all moderate in strength). Among older male patients, PMI was positively correlated with lipase (moderate), while PvMI was negatively correlated with the Charlson Comorbidity Index (CCI) (moderate). No other statistically significant correlations were identified between PMI or PvMI and the remaining biochemical parameters (all *p* > 0.05).

All statistically significant correlations between PMI or PvMI and clinical–biochemical parameters, observed in the overall cohort and in the sex-based and age-based (≥65 years) subgroups, are summarized in Table 2.

### 3.5. Association of Muscle Indices with Complicated Disease Course and Intensive Care Requirement

In female patients, logistic regression analysis demonstrated a significant association between PMI and the development of complicated pancreatitis. While the univariate analysis showed a trend toward statistical significance (OR = 0.854; *p* = 0.063), the multivariate model indicated that PMI was an independent factor associated with a complicated disease course (OR = 0.655; *p* = 0.018). In patients aged 65 years and older, PvMI was identified as an independent parameter associated with the need for intensive care in both the univariate (OR = 0.816; *p* = 0.035) and adjusted models (OR = 0.780; *p* = 0.047). In addition, PMI appeared to be an independent factor associated with the development of complicated pancreatitis in the adjusted model (OR = 0.775; *p* = 0.045). The results of univariate and multivariate logistic regression analyses examining the associations between muscle indices and both the development of complicated pancreatitis and the need for intensive care are presented in Table 3.

### 3.6. Comparisons of Muscle Indices According to Clinical Outcomes in Patients Aged 65 Years and Older

**Figure 2:**
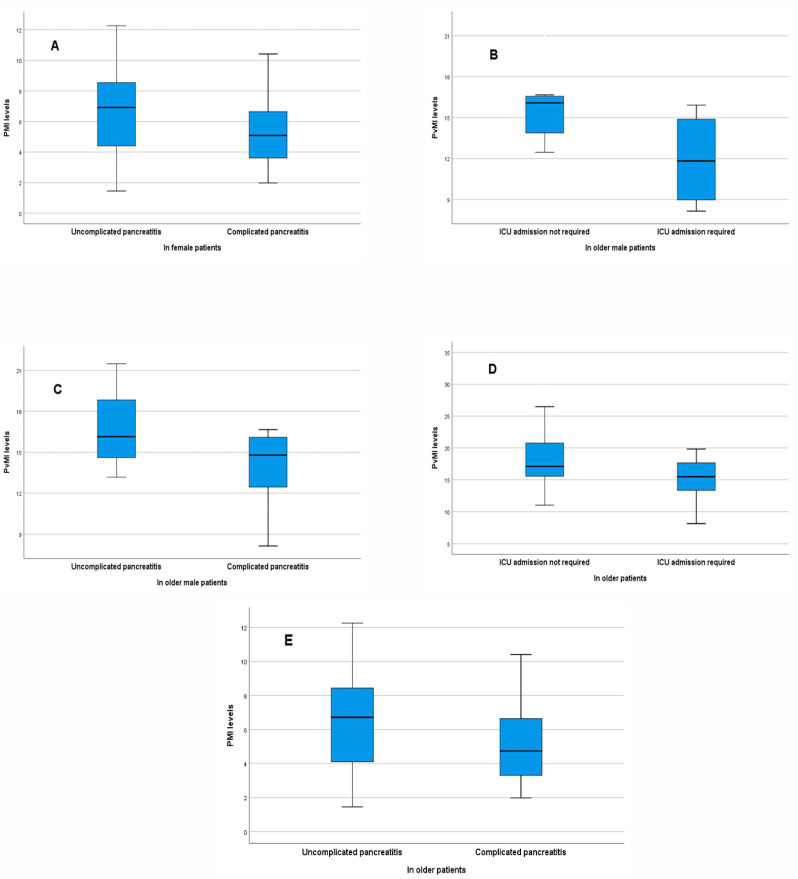
Comparisons of Muscle Indices According to Complication Status and Intensive Care Requirement in Different Subgroups. (**A**) Among all female patients, the median PMI value was lower in those with complicated pancreatitis (5.09 cm^2^/m^2^; range: 1.98–10.41) than in those without complications (6.92 cm^2^/m^2^; range: 1.45–12.26), with borderline significance (*p* = 0.072). (**B**) In male patients aged ≥65 years, the mean PvMI value was lower in those requiring ICU admission (11.93 cm^2^/m^2^; SD: 3.58) compared with those not requiring ICU (16.05 cm^2^/m^2^; SD: 2.61) (*p* = 0.020). (**C**) In male patients aged ≥65 years, the mean PvMI was lower in those with complicated pancreatitis (13.86 cm^2^/m^2^; SD: 2.94) than in those without complications (16.73 cm^2^/m^2^; SD: 3.05), with borderline significance (*p* = 0.059). (**D**) In patients aged ≥65 years, the mean PvMI value was lower in those requiring ICU admission (15.05 cm^2^/m^2^; SD: 3.50) compared with those not requiring ICU (18.03 cm^2^/m^2^; SD: 4.28) (*p* = 0.029). (**E**) In patients aged ≥65 years, the median PMI was lower in those with complicated pancreatitis (4.74 cm^2^/m^2^; range: 1.98–10.41) than in those without complications (6.72 cm^2^/m^2^; range: 1.45–12.26), with borderline significance (*p* = 0.053).

**Figure 3:**
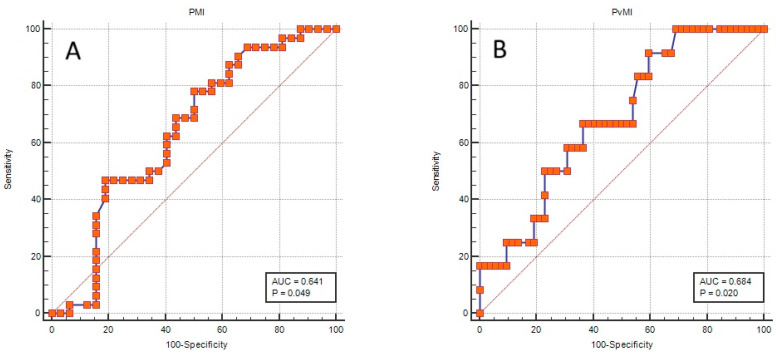
ROC curves of muscle indices for predicting complicated pancreatitis and intensive care requirement in patients aged 65 years and older. (**A**) The optimal cutoff value of PMI for predicting complicated pancreatitis was identified as ≤4.04 cm^2^/m^2^ (Youden index: 0.281; AUC: 0.641; *p* = 0.049; Sensitivity: 47%; Specificity: 81%). (**B**) The optimal cutoff value of PvMI for predicting ICU requirement was identified as ≤18.88 cm^2^/m^2^ (Youden index: 0.321; AUC: 0.684; *p* = 0.020; Sensitivity: 92%; Specificity: 40%).

In patients aged 65 years and older, the mean PvMI was significantly lower in those who required intensive care (15.05 ± 3.50 cm^2^/m^2^) than in those who did not (18.03 ± 4.28 cm^2^/m^2^; *p* = 0.029). Among male patients aged 65 years and older, the mean PvMI was also significantly lower in those requiring intensive care (11.93 ± 3.58 cm^2^/m^2^) than in those who did not (16.05 ± 2.61 cm^2^/m^2^; *p* = 0.020). The analyses evaluating the associations of muscle indices with the development of complications and the need for intensive care, stratified by age and sex, are illustrated in Figure 2. In this age group, the optimal cutoff value for PMI in predicting complicated pancreatitis was ≤4.04 cm^2^/m^2^ (AUC = 0.641, *p* = 0.049). The optimal cutoff value of PvMI for predicting the requirement for intensive care was identified as ≤18.88 cm^2^/m^2^ (AUC = 0.684, *p* = 0.020) (Figure 3).

## 4. Discussion

This study evaluated the potential impact of psoas and paravertebral muscle indices, measured using computed tomography, on the clinical course of acute pancreatitis. Their associations with the development of complications and the requirement for intensive care were also assessed. The findings suggest that these muscle indices may show exploratory associations with disease severity and ICU requirements rather than serving as definitive prognostic indicators, particularly among older patients, in whom reduced muscle indices were linked to a more severe disease course and a higher likelihood of requiring intensive care. However, these associations were not observed in the overall cohort and should therefore be interpreted as exploratory.

Multiple factors influence the clinical course of acute pancreatitis. While mild cases are usually self-limiting, severe forms may progress with pancreatic necrosis, infection, and multiorgan failure, leading to mortality rates as high as 10–30%. Previous studies have shown that advanced age, a high comorbidity burden, hypoproteinemia, elevated CRP and WBC levels, and hypocalcemia are among the major factors associated with increased mortality [15]. Consistent with the existing literature, our study also demonstrated that patients who developed complications or required intensive care were characterized by higher age, CCI scores, and inflammatory markers, as well as lower calcium and albumin levels.

Previous studies have demonstrated that sarcopenia is associated with elevated inflammatory markers and reduced nutritional parameters [5]. Similarly, in our study, lower muscle mass indices were correlated with higher CRP, WBC, and neutrophil counts, as well as with lower albumin and hemoglobin levels. These findings suggest that loss of muscle mass reflects not only nutritional depletion but also an increased inflammatory burden, which may contribute to adverse outcomes in patients with acute pancreatitis. In line with this, several studies have reported associations between sarcopenia and adverse clinical outcomes in acute pancreatitis, including prolonged hospitalization, higher complication rates, and increased mortality [16,17].

In recent years, the incidence of acute pancreatitis has markedly increased among older adults, in parallel with the aging of the general population. It is well documented that acute pancreatitis tends to follow a more severe course in this age group. Systematic reviews and multicenter studies have consistently demonstrated that complication rates, organ failure, and mortality are higher in patients aged 65 years and older compared with younger adults. The higher comorbidity burden, increased frailty, and atypical clinical presentations in older patients may lead to delays in diagnosis and management, thereby further increasing the risk of complications and mortality in this population [18,19,20]. In our study, patients who developed complications or required intensive care had a higher mean age across the entire cohort. In light of these findings, the identification and clinical integration of additional markers that can predict complications and the need for intensive care—particularly in older adults with acute pancreatitis—are of great importance. Previous studies have predominantly demonstrated the association between sarcopenia and adverse clinical outcomes of acute pancreatitis in cohorts consisting of older adults and patients with a high comorbidity burden. While the independent prognostic value of sarcopenia in younger adults has not yet been clearly established [9], our study similarly found no significant association between muscle indices and either complication development or intensive care requirement in the overall cohort. However, in subgroup analyses of older adults, lower PvMI values appeared to be associated with the need for intensive care, although these subgroup findings should be considered hypothesis-generating rather than definitive. In this subgroup, the ROC analyses demonstrated that PMI and PvMI exhibited only modest discriminatory performance for predicting complication development and ICU requirement. For PMI, an AUC of 0.641 with a sensitivity of 47% and specificity of 81% indicates that lower PMI values have limited sensitivity but relatively higher specificity, suggesting a restricted yet potentially supportive role in identifying older patients at increased risk for complicated disease. For PvMI, an AUC of 0.684 combined with high sensitivity (92%) but low specificity (40%) suggests that reduced PvMI may function as a sensitive early screening indicator for ICU requirement, although its low specificity limits its utility as a standalone prognostic tool. Accordingly, PMI and PvMI should not be considered strong independent predictors in older adults but rather complementary markers that may provide additional risk signals alongside established clinical and biochemical parameters. Given their modest discriminative ability, these findings remain exploratory and require validation in larger, prospective cohorts.

Another important finding of our study was that, in the overall cohort, lower PMI values in female patients were associated with the development of complicated pancreatitis. In contrast, higher PMI values appeared to be independent protective factors against a complicated disease course. Nevertheless, these associations were limited to subgroup analyses and were not present in the whole cohort; therefore, they should be interpreted as preliminary and exploratory. However, the role of low PMI or PvMI values—particularly in women and in patients aged 65 years and older—in predicting complicated pancreatitis or the need for intensive care has not yet been investigated explicitly in sufficient detail in the existing literature. The present results, therefore, suggest that muscle indices may have subgroup-specific associations. Moreover, the observed associations between lower PMI and PvMI values and adverse clinical outcomes in these subgroups may suggest that reduced muscle mass contributes to a more severe disease course through both nutritional and inflammatory mechanisms.

One of the significant strengths of this study is the systematic evaluation of the potential effects of both psoas and paravertebral muscle indices on the clinical course, the development of complications, and the need for intensive care within the same cohort of patients with acute pancreatitis. In addition, the separate assessment of muscle indices across age- and sex-based subgroups identified novel findings with possible prognostic relevance, particularly among older and female patients. The comprehensive analysis of clinical, biochemical, and muscle-related parameters using multiple statistical approaches enabled an integrated evaluation of muscle loss and inflammatory burden, while also providing indirect insight into the patients’ nutritional status, thereby enhancing the robustness and clinical interpretability of the findings.

This study has several limitations. The retrospective, single-center design, along with the relatively small sample size, may limit the generalizability of the findings. In addition, because direct assessments of muscle strength were unavailable, the evaluation of sarcopenia relied solely on muscle mass measurements. The absence of long-term mortality data and the very low mortality rate observed in the current cohort also limited the ability to assess the prognostic impact of muscle indices on mortality. The heterogeneity of findings reported in the literature regarding the predictive value of muscle indices may be attributed to differences in study populations, measurement techniques, comorbidities, and cutoff definitions [9]. In this context, it should be noted that the cutoff values identified in the present study through ROC analysis were derived from the same cohort and have not yet been externally validated. Moreover, the discriminatory performance of PMI and PvMI in predicting complications or ICU requirement was modest. Therefore, further studies are needed to confirm the external validity of these thresholds and to establish standardized reference values applicable to different patient populations.

## 5. Conclusions

This study suggests that psoas and paravertebral muscle indices may be associated with the clinical course and adverse outcomes in patients with acute pancreatitis. Particularly among older and female patients, lower muscle indices showed exploratory associations that may indicate potential prognostic value for predicting complications and the need for intensive care. However, these findings were not observed in the overall cohort, and the indices’ discriminatory performance remained modest. Therefore, the observed associations should be interpreted as exploratory. Incorporating these parameters into clinical practice should be considered only after further validation, as larger studies are needed to confirm their prognostic utility. Thus, external validation in larger, prospective multicenter cohorts will be essential before these markers can be recommended for clinical implementation.

## Figures and Tables

**Table 1:** General characteristics of patients and comparisons by complication status and ICU requirement.

Parameters	All Patients n = 179	Non-Complicated AP n = 108	Complicated AP n = 71	*p*-Value	Without ICU Admission n = 159	With ICU Admission n = 20	*p*-Value
*General Characteristics*							
• Age, years	58 (19–97)	54 (19–97)	63 (21–90)	0.006	57 (19–97)	68 (26–90)	0.005
• Gender, female	95 (53.1)	59 (54.6)	36 (50.7)	0.607	85 (53.5)	10 (50.0)	0.770
• BMI, kg/m^2^	25.71 (19.05–44.62)	25.84 (19.05–44.62)	25.71 (20.55–38.06)	0.892	25.71 (19.05–44.62)	25.69 (20.55–37.87)	0.491
• Length of stay, days	7 (3–40)	6 (4–13)	10 (3–40)	<0.001	7 (4–24)	13 (3–40)	<0.001
• Number of AP attacks	1 (1–5)	1 (1–5)	1 (1–3)	0.859	1 (1–5)	1 (1–5)	0.009
• CCI	0 (0–4)	0 (0–3)	0 (0–4)	0.115	0 (0–3)	1 (0–4)	<0.001
*Etiology*							
• Non-biliary	108 (60.3)	72 (66.7)	36 (50.7)	0.033	97 (61.0)	11 (55.0)	0.605
• Biliary	71 (39.7)	36 (33.3)	35 (49.3)		62 (39.0)	9 (45.0)	
*Disease severity*							
• Mild	108 (60.3)	102 (94.4)	6 (8.5)	<0.001	106 (66.7)	2 (10.0)	<0.001
• Moderate	54 (30.2)	5 (4.6)	49 (69.0)		47 (29.6)	7 (35.0)	
• Severe	17 (9.5)	1 (0.9)	16 (22.5)		6 (3.8)	11 (55.0)	
ICU need	20 (11.2)	3 (2.8)	17 (23.9)	<0.001			
Complication rate	71 (39.7)	-	-	-	54 (34.0)	17 (85.0)	<0.001
In-hospital mortality	3 (1.7)	0 (0.0)	3 (4.2)	0.061	0 (0.0)	3 (15.0)	<0.001
Muscle measurements							
• PMI, cm^2^/m^2^	5.51 (1.45–12.26)	5.53 (1.45–12.26)	5.51 (1.98–10.73)	0.331	5.51 (1.45–12.26)	5.74 (2.29–9.77)	0.608
• PvMI, cm^2^/m^2^	16.9 ± 4.12	17.07 ± 4.05	16.66 ± 4.24	0.514	17 ± 4.11	16.17 ± 4.22	0.400
• Patients with low PMA, n (%)	43 (24)	28 (25.9)	15 (21.1)	0.462	39 (24.5)	4 (20.0)	0.786
• Patients with low PvMA, n (%)	67 (37.4)	38 (35.2)	29 (40.8)	0.444	59 (37.1)	8 (40.0)	0.801
*Laboratory values*							
• WBC, 10^3^/µL	10.75 (2.1–39.54)	10.17 (3.3–21.61)	13.33 (2.1–39.54)	<0.001	10.62 (2.1–21.61)	19.31 (6–39.54)	<0.001
• PLT, 10^3^/µL	251 (85–605)	249 (100–434)	251 (85–605)	0.378	249 (85–495)	257 (100–605)	0.636
• HB, g/dL	13.94 ± 2.08	13.74 ± 1.99	14.23 ± 2.19	0.125	13.94 ± 2.03	13.95 ± 2.51	0.985
• ALT, U/L	43 (5–1498)	34 (5–696)	58 (9–1498)	0.144	37 (5–729)	120.5 (19–1498)	0.015
• AST, U/L	46 (5–3647)	36 (5–1612)	75 (5–3647)	0.169	38 (5–1612)	191.5 (17–3647)	0.005
• ALP, U/L	93 (32–663)	89 (32–631)	109 (40–663)	0.145	90 (32–631)	141 (44–663)	0.010
• GGT, U/L	65 (4–1542)	50 (4–1204)	133 (8–1542)	0.023	55 (4–1204)	225 (14–1542)	0.006
• Amylase, U/L	625 (8–4618)	473.5 (8–4618)	884 (28–4228)	0.029	603 (8–4618)	793.5 (28–4228)	0.716
• Lipase, U/L	1291 (15–12,701)	1165 (24–12,701)	1458 (15–10,204)	0.098	1262 (24–12,701)	1423.5 (15–9461)	0.887
• Glucose, mg/dL	118 (54–474)	107 (58–474)	131 (54–406)	<0.001	116 (54–474)	149 (60–406)	0.050
• Creatinine, mg/dL	0.8 (0.39–3.9)	0.76 (0.41–3.9)	0.85 (0.39–2.48)	0.053	0.77 (0.41–3.9)	1 (0.39–2.48)	<0.001
• Total Bilirubin, mg/dL	0.7 (0.14–7)	0.59 (0.14–5)	0.85 (0.17–7)	0.014	0.64 (0.14–7)	1.05 (0.37–6.92)	0.015
• Direct Bilirubin, mg/dL	0.23 (0.09–4.96)	0.2 (0.09–3.14)	0.3 (0.09–4.96)	0.038	0.21 (0.09–4.96)	0.46 (0.09–4.63)	0.024
• Albumin, g/L	40 (19–49)	41 (27–49)	40 (19–49)	0.338	41 (24–49)	37.5 (19–46)	0.018
• Calcium, mg/dL	8.6 (6.3–10.7)	8.6 (6.9–9.9)	8.5 (6.3–10.7)	0.442	8.6 (6.8–10.7)	8.25 (6.3–10.4)	0.038
• Triglyceride, mg/dL	104 (38–3421)	100 (38–3421)	120 (38–1257)	0.028	101 (38–3421)	158 (55–1256)	0.050
• CRP, mg/L	15.3 (0.6–290)	12 (0.6–105)	23.42 (0.6–290)	0.003	13.76 (0.6–290)	47.19 (4.6–247)	0.002
• Procalcitonin, µg/L	0.1 (0.01–100)	0.06 (0.01–92)	0.38 (0.02–34.8)	<0.001	0.08 (0.01–42)	3 (0.12–100)	<0.001

BMI: Body mass index; CCI: Charlson comorbidity index; PMI: Psoas muscle index; PvMI: Paravertebral muscle index; WBC: White blood cell; PLT: Platelet; HB: Hemoglobin; ALT: Alanine aminotransferase; AST: Aspartate aminotransferase; ALP: Alkaline phosphate; GGT: Gama glutamyl transferase; CRP: C-reactive protein; ICU: Intensive care unit.

**Table 2:** Parameters showing statistically significant correlations with PMI and PvMI in the overall and subgroup analyses.

Parameters	Rho	*p*-Value
***All patients***		
• PMI vs. Albumin	0.180	0.016
• PMI vs. CRP	−0.160	0.032
• PvMI vs. WBC	−0.156	0.037
• PvMI vs. Hemoglobin	−0.270	<0.001
• PvMI vs. Creatinine	−0.257	0.001
***Female patients***		
• PMI vs. WBC	−0.307	0.002
• PMI vs. NEU	−0.311	0.002
• PMI vs. Albumin	0.207	0.044
• PMI vs. Triglyceride	−0.251	0.032
• PvMI vs. ALT	−0.204	0.048
• PvMI vs. GGT	−0.274	0.007
• PvMI vs. Creatinine	−0.217	0.035
***Male patients***		
• PMI vs. Amylase	0.258	0.018
• PMI vs. Lipase	0.284	0.009
• PMI vs. Glucose	0.233	0.042
• PMI vs. CRP	−0.253	0.020
• PvMI vs. Amylase	0.268	0.014
• PvMI vs. Lipase	0.281	0.009
• PvMI vs. ALP	0.280	0.010
***≥65 years (all patients)***		
• PvMI vs. Creatinine	−0.390	0.001
• PvMI vs. ALT	−0.249	0.047
***≥65 years (female patients)***		
• PvMI vs. Creatinine	−0.319	0.031
• PvMI vs. GGT	−0.392	0.007
• PvMI vs. AST	−0.316	0.032
• PvMI vs. ALT	−0.335	0.023
***≥65 years (male patients)***		
• PMI vs. Lipase	0.490	0.039
• PvMI vs. CCI	−0.479	0.044

PMI: Psoas muscle index; CRP: C-reactive protein; WBC: White blood cell; NEU: Neutrophil; ALT: Alanine aminotransferase; GGT: Gamma-glutamyl transferase; ALP: Alkaline phosphatase; PvMI: Paravertebral muscle index; CCI: Charlson comorbidity index. Note: Correlations of all numerical parameters with PMI and PvMI were examined and only statistically significant results are presented in the table.

**Table 3:** Univariate and multivariate logistic regression analyses of muscle indices associated with complicated pancreatitis and intensive care requirement.

Outcome	Subgroup	Muscle Index	OR	95% CI	*p*-Value
**Complicated pancreatitis**	Female patients	PMI ^1^	0.854	0.723–1.009	0.063
		PMI ^2^	0.655	0.462–0.929	0.018
	≥65 years (male patients)	PvMI ^3^	0.669	0.411–1.088	0.105
	≥65 years (all patients)	PMI ^4^	0.829	0.682–1.007	0.059
		PMI ^5^	0.775	0.604–0.995	0.045
**ICU requirement**	≥65 years (male patients)	PvMI ^6^	0.548	0.291–1.032	0.063
	≥65 years (all patients)	PvMI ^7^	0.816	0.675–0.986	0.035
		PvMI ^8^	0.780	0.611–0.997	0.047

PMI: Psoas muscle index; PvMI: Paravertebral muscle index; ICU: Intensive care unit. Notes: ^1^, ^3^, ^4^, ^6^, ^7^: Univariate regression. ^2^: Adjusted regression (adjusted for age, neutrophil, lymphocyte, GGT, glucose, CRP, length of hospital stay, total bilirubin, etiology, and triglyceride levels). ^5^: Adjusted regression (adjusted for age, sex, length of hospital stay, WBC, neutrophil, and platelet counts). ^8^: Adjusted regression (adjusted for age, CCI, ALP, GGT, and etiology).

## Data Availability

The datasets generated during and/or analyzed during the current study are available from the corresponding author on reasonable request.

## Supplementary Materials

The following supporting information can be downloaded at https://www.mdpi.com/article/10.3390/medicina62010054/s1, Supplementary Table S1. Modified Charlson Comorbidity Index (CCI) Components and Scoring System.

## Abbreviations

The following abbreviations are used in this manuscript:

AP	Acute pancreatitis
ALT	Alanine aminotransferase
AST	Aspartate aminotransferase
ALP	Alkaline phosphatase
BISAP	Bedside Index for Severity in Acute Pancreatitis
CCI	Charlson Comorbidity Index
CRP	C-reactive protein
CT	Computed tomography
GGT	Gamma-glutamyl transferase
ICU	Intensive care unit
L3	Third lumbar vertebral level
PMI	Psoas muscle index
PvMI	Paravertebral muscle index
WBC	White blood cell
