# Supplementary material for: Potential Associations Between CT-Derived Muscle Indices and Clinical Outcomes in Acute Pancreatitis
Source: Medicina (Kaunas). 2025 Dec 27;62(1):54. doi: 10.3390/medicina62010054 (PMC12842836; doi:10.3390/medicina62010054)
Supplement: Supplementary file 1 [file medicina-62-00054-s001.zip › medicina-3946068-supplementary.pdf]

Supplementary Table S1. Modified Charlson Comorbidity Index (CCI) Components and Scoring System

Comorbidity	Score
Myocardial infarction	0
Congestive heart failure	2
Peripheral vascular disease	0
Cerebrovascular disease	0
Dementia	2
Chronic pulmonary disease	1
Rheumatologic disease	1
Peptic ulcer disease	0
Mild liver disease	2
Diabetes without chronic complications	0
Diabetes with chronic complications	1
Hemiplegia or paraplegia	2
Renal disease	1
Any malignancy, including leukemia and lymphoma	2
Moderate or severe liver disease	4
Metastatic solid tumor	6
AIDS/HIV	4
Maximum comorbidity score	24
